# Targeting Leukemia-Initiating Cells and Leukemic Niches: The Next Therapy Station for T-Cell Acute Lymphoblastic Leukemia?

**DOI:** 10.3390/cancers14225655

**Published:** 2022-11-17

**Authors:** Ziting Zhang, Kun Yang, Han Zhang

**Affiliations:** 1 Institute of Medical Biology, Chinese Academy of Medical Sciences and Peking Union Medical College, Kunming 650118, China; 2 School of Life Sciences, Yunnan University, Kunming 650500, China

**Keywords:** T-cell acute lymphoblastic leukemia, leukemia-initiating cell, microenvironment, leukemic niche, precision medicine, small-molecule inhibitor, monoclonal antibody

## Abstract

**Simple Summary:**

T-cell acute lymphoblastic leukemia (T-ALL) is an aggressive malignancy arising from the aberrant proliferation of immature T-cell progenitors. Despite improved insights in genetic and biological characteristics of T-ALL, clinical therapy has remained largely similar. Recent studies have shown that leukemia-initiating cells (LICs) and leukemic niches play major roles in the initiation and progression of T-ALL, thus, facilitating the development of targeted therapies. This review provides a broad overview of the recent discoveries on LICs and leukemic niches in the context of T-ALL, with a particular focus on the current precision medicine.

**Abstract:**

T-cell acute lymphoblastic leukemia (T-ALL) is an aggressive subtype of hematological malignancy characterized by its high heterogeneity and potentially life-threatening clinical features. Despite the advances in risk stratification and therapeutic management of T-ALL, patients often suffer from treatment failure and chemotherapy-induced toxicity, calling for greater efforts to improve therapeutic efficacy and safety in the treatment of T-ALL. During the past decades, increasing evidence has shown the indispensable effects of leukemia-initiating cells (LICs) and leukemic niches on T-ALL initiation and progression. These milestones greatly facilitate precision medicine by interfering with the pathways that are associated with LICs and leukemic niches or by targeting themselves directly. Most of these novel agents, either alone or in combination with conventional chemotherapy, have shown promising preclinical results, facilitating them to be further evaluated under clinical trials. In this review, we summarize the latest discoveries in LICs and leukemic niches in terms of T-ALL, with a particular highlight on the current precision medicine. The challenges and future prospects are also discussed.

## 1. Introduction

T-cell acute lymphoblastic leukemia (T-ALL) is an aggressive subtype of ALL characterized by the clonal proliferation of immature T-cell precursors, accounting for approximately 15% of pediatric and 25% of adult ALL cases [1]. Patients with T-ALL often present with signs and symptoms of high leukocyte count that is linked to the direct T-ALL infiltration to the bone marrow (BM), extramedullary infiltration towards the central nervous system (CNS), and hematopoietic failure that is related to the decreased production of BM elements. Despite the remarkable improvement in risk classification based on the distinct immunophenotypes and gene expression profiles [2], T-ALL patients often suffer from chemoresistance, resulting in treatment failure and disease relapse. Chemotherapy-induced toxicity is another great challenge that cannot be overlooked. Therefore, it is urgent to develop innovative and safe therapeutic strategies that rely primarily on a deeper understanding of the biological underpinnings of T-ALL.

Over the past decades, increasing evidence has shown that T-ALL development in animal models is initiated by a rare population of cells called leukemia-initiating cells (LICs) [3]. The dynamic interplay between leukemic cells and their microenvironments (e.g., leukemic niches) also plays an indispensable role in the development of T-ALL. These discoveries facilitate precision medicine for T-ALL by targeting either the aberrant pathways that regulate LICs and leukemic niches or themselves. Although the molecular mechanisms underlying the intrinsic nature of LICs and the microenvironmental landscape of T-ALL remain ill-defined, some novel agents have shown promising efficacy and acceptable toxicity in patients with T-ALL. As such, we are now at a crossroad in terms of the future treatment of T-ALL: What is the next therapy station? To continue the contemporary intensive multiagent chemotherapy? Or to specifically target LICs or leukemic niches as initial therapy? Or both? To answer these questions, we summarized the recent discoveries on LICs and leukemic niches, and discussed current insights into the precision medicine for T-ALL.

## 2. LICs in T-ALL

LICs, also termed leukemia stem cells (LSCs), were first identified by Dick and his colleagues in studies on acute myeloid leukemia (AML) [4], characterized by their self-renewal capability and potential to differentiate into leukemic blasts [5,6]. Although LICs and LSCs are used interchangeably for AML, their concepts are not necessarily the same [7]. The LICs more appropriately denote the leukemia cells of origin, whereas the LSCs refer to a distinct subpopulation with the capacity for self-renewal and long-term clonal maintenance at a later stage [6,7]. In this context, the LICs are at the apex of the leukemic hierarchy, whereas the LSCs represent cells that can be prospectively isolated from the remainder of the cancer cells based on specific cell surface markers. However, in some cancers such as T-ALL, it is not possible to distinguish LSCs from non-LSCs due to the ill-defined immunophenotypes. In this regard, Dick and others have defined such cells as LICs by their ability to (i) generate leukemia in transplanted xenografts, (ii) self-renew upon serial passages in xenografts, and (iii) give rise to daughter cells with proliferative capacity but that are unable to maintain the tumor clone after serial passages [6]. Nonetheless, the definition of LICs in T-ALL are not well characterized. In most instances, the T-ALL LICs (T-LICs) and LSCs are still used interchangeably, whereas in some studies, the T-LICs only refer to the transplanted cells with leukemia-initiating capacity. As such, we use a unified term of T-LICs to refer to all cells with leukemia-initiating potential and/or LSC capacity.

### 2.1. T-LICs in Mouse Models

T-LICs were mostly studied in genetically engineered mouse models of T-ALL, among which, the T-cell acute lymphocytic 1 (TAL) basic helix-loop-helix (bHLH) transcription factor 1 (*Tal1*)-induced mouse model stands out, since 28% of the *Tal1* transgenic mice develop leukemia [8]. Additionally, co-expression of *Tal1* with LIM domain only 1 (*Lmo1*) or *Lmo2* not only accelerates T-ALL onset and progression [9,10] but also provides a favorable context for the acquisition of activating mutations of notch receptor 1 (*NOTCH1*) and the emergence of T-LICs [11]. Thus, the *Tal1-Lmo1/2* transgenic mouse is commonly utilized as a model for characterizing T-LICs [11,12]. Moreover, the retroviral or transgenic mouse models of *Notch1*-induced T-ALL [13,14,15,16,17], KRAS proto-oncogene (*Kras*)*^G12D^*-induced T-ALL [13,18], and phosphatase and tensin homolog (*Pten*)-null T-ALL [19,20] are also used for identification of T-LICs in terms of different T-ALL-causing mutations.

**Figure 1:**
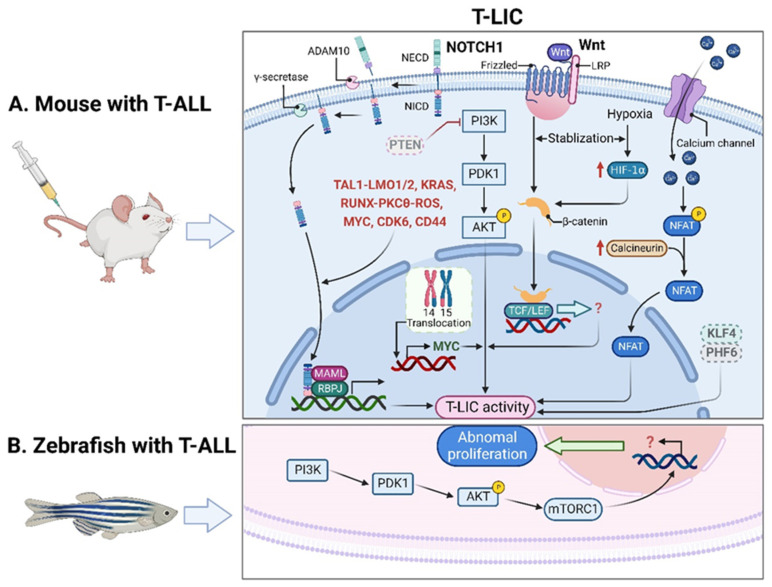
T-LICs in animal models. (**A**) The formation and maintenance of T-LICs involve multiple genetic and molecular events, independent or in cooperation with NOTCH1. Briefly, NOTCH1 acts as a ligand-activated transcription factor. The ligand–receptor interaction triggers the cleavage of the NECD by ADAM10 metalloproteases, followed by the subsequent NICD cleavage by γ-secretases. Once released from the membrane, the cytoplasmic NICD translocates into the nucleus, where it activates gene transcription via association with RBPJ DNA-binding protein and MAML transcriptional co-activator. NOTCH1 also modulates T-LIC activity by cooperating with TAL1-LMO1/2, KRAS, RUNX-mediated PKCθ and ROS, MYC, CDK6, or CD44. On the other hand, inactivation of PTEN-induced PI3K-AKT activation, together with the subsequent activation of β-catenin, as well as the aberrant overexpression of MYC induced by t (14;15) translocation, contributes to the onset of T-ALL independent of NOTCH1. Specifically, the binding of Wnt ligand to its receptor Frizzled and/or LRP leads to the activation of Wnt signaling, which stabilizes β-catenin from degradation. Further translocation of β-catenin into the nucleus promotes the downstream gene transcription by binding to TCF/LEF. Finally, the maintenance of T-LIC activity also requires active HIF1α protein, which supports Wnt signaling by promoting accumulation of β-catenin proteins. Activation of calcineurin/NFAT signaling is also critical for T-LIC activity, and the same holds true for the inactivation of KLF4 or PHF6. (**B**) The increased frequency of T-LICs in zebrafish models might be caused by the abnormal activation of AKT-mTORC1 signaling. ADAM10, a disintegrin and metalloprotease 10; AKT, AKT serine/threonine kinase; CDK6, cyclin dependent kinase 6; HIF1α, hypoxia-induced factor 1 alpha; KLF4, Krüppel-like factor 4; KRAS, KRAS proto-oncogene; LEF, lymphoid enhancer-binding factor; LMO1/2, LIM domain only 1/2; LRP, lipoprotein-receptor-related protein; MAML, mastermind-like; mTORC1, mechanistic target of rapamycin kinase complex 1; MYC, MYC proto-oncogene; NECD, NOTCH extracellular domain; NFAT, nuclear factor of activated T cells; NICD, NOTCH intracellular domain; NOTCH1, notch receptor 1; PHF6, plant homeodomain factor 6; PI3K, phosphatidylinositol 3-kinase; PKCθ, protein kinase C theta; PTEN, phosphatase and tensin homolog; RBPJ, recombination signal binding protein for immunoglobulin kappa J region; ROS, reactive oxygen species; RUNX, runt-related transcription factor; TAL1, T-cell acute lymphocytic 1 (TAL) basic helix-loop-helix (bHLH) transcription factor 1; T-ALL, T-cell acute lymphoblastic leukemia; TCF, T-cell factor; T-LICs, T-ALL leukemia-initiating cells; Wnt, Wingless/Integrated.

Of note, the genes that encode TAL1 and LMO1/2 are recurring targets of chromosomal translocation [21], and the activating mutations of *NOTCH1* were identified in more than 60% of human T-ALL cases [22,23]. Interestingly, NOTCH1 was revealed as a key regulator of human T-LIC activity, since inhibition of the NOTCH1 pathway by γ-secretase inhibitors (GSIs) abolishes T-LIC activity in xenografts and mouse models [12,24,25]. However, the gain-of-function mutations in *NOTCH1* can initiate T-ALL in mouse models but have weak leukemogenic strength, implying that additional cooperating events are required [13]. Indeed, NOTCH1 modulates T-LIC activity by cooperating with oncogenic TAL1-LMO1/2 transcription factors [11], KRAS [13,18], runt-related transcription factor (RUNX)-mediated regulation of protein kinase C theta (PKCθ) and reactive oxygen species (ROS) [14], MYC proto-oncogene (MYC) [16], cyclin dependent kinase 6 (CDK6) [17], or CD44 [26] (Figure 1A).

In addition, other signals contribute to the onset of T-ALL independent of NOTCH1 signaling. For instance, the loss of PTEN and the activation of the phosphatidylinositol 3-kinase (PI3K) pathway were implicated in human T-ALL samples [27,28,29,30]. Mechanistically, *Pten* deletion-induced PI3K-AKT serine/threonine kinase (AKT) activation offers the first hit in hematopoietic stem cells (HSCs); the subsequent activation of β-catenin contributes to the acquisition of the self-renewal capacity in T-LICs; a recurring t (14;15) translocation further results in aberrant overexpression of *Myc*, ultimately promoting the formation of *Pten*-null T-LICs [19]. This finding provides strong evidence that at least two mutational events are required to generate leukemia: one transforming HSCs and/or hematopoietic progenitor cells (HPCs) into LICs and a second triggering full-blown leukemia. Additionally, the maintenance of T-LIC activity and the expansion of T-LICs also require the activation of calcineurin [31] or hypoxia inducible factor 1α (HIF1α) [32] or the inactivation of Krüppel-like factor 4 (KLF4) [33] or plant homeodomain factor 6 (PHF6) [34]. To sum up, the formation and maintenance of T-LICs involve various genetic and molecular events, either independent or in cooperation with NOTCH1 (Figure 1A).

Despite the above, the differentiation hierarchy and immunophenotypic identification of T-LICs are highly variable between models, making their prospective isolation difficult. In *Tal1-Lmo1* transgenic mice, T-LICs are found in both the CD4^−^CD8^−^ double-negative (DN) and CD4^+^CD8^+^ double-positive (DP) populations in the thymus, even though the leukemic DN3 and DN4 populations have the highest leukemia-initiating potential [11]. Likewise, in the *Tal1-Lmo2* transgenic mouse models, the T-LIC potential is mainly enriched in the DN3 cells [12], and the same is true in the *Lmo2* transgenic mice [35]. In contrast, the CD8^+^CD4^−^HSA^hi^ single-positive subset but not the DN or DP population develops T-ALL in *Notch1*-induced mouse models [15], whereas in the *Pten*-null T-ALL models, T-LICs are enriched in the c-Kit^mid^CD3^+^ compartment [19]. These findings suggest that the types of T-LICs are highly dependent on the genetic alterations. In addition, the identity of T-LICs in different age groups is also being debated [15,36,37,38,39]. For example, CD34 was reported as a marker for T-LICs in xenografts from pediatric T-ALL [36], whereas in adult T-ALL-derived xenografts, the CD7^+^CD1a^−^ but not the CD34^+^ subset initiates leukemia in response to NOTCH1 activation [37]. Such a discrepancy reflects greater biological heterogeneity of T-LICs, which calls for further refinement of their nature and function in different age-defined groups.

### 2.2. T-LICs in Zebrafish Models

In addition to the studies in mouse xenografts, zebrafish were also employed to recapitulate human T-ALL. Interestingly, leukemic cells from zebrafish models can be transplanted serially, suggesting the presence of T-LICs [40]. Unlike a minor subpopulation of T-LICs in human and murine T-ALL, the T-LICs are abundant in the *Myc*-induced T-ALL zebrafish models based on large-scale single-cell transplantation experiments [41]. One possible reason might be due to the abnormal activation of AKT-mechanistic target of rapamycin kinase complex 1 (mTORC1) signaling, which enhances the overall frequency of T-LICs in zebrafish [42] (Figure 1B).

### 2.3. Therapies Targeting T-LICs

Currently, the difficulty in the treatment of T-ALL is that the conventional therapeutics mainly target the bulk of leukemic cells but not the T-LICs. In this context, strategies that eradicate T-LICs, the main culprit for relapse, may have significant clinical implications. The above discoveries of T-LICs in animal models pave a new way to target T-LICs by interfering with the pathways that regulate T-LIC activity. For example, the therapies that target NOTCH1 signaling [12,24], PI3K-AKT pathway [20,43], MYC [20,44], calcineurin [45] or CD44 [46] can eliminate T-LICs or impair their activities. In addition, the existing therapeutic agents approved for other indications are also identified to eliminate T-LICs. For example, metformin, an anti-diabetic drug, could induce apoptosis of LIC-enriched T-ALL cells, but the mechanism underlying the metformin action remains undetermined [47]. Parthenolide, an anti-inflammatory agent, can also induce apoptosis of T-LICs, but the individual T-LIC subpopulations within patients have different responses to parthenolide, as disease progression is slowed but not prevented in xenografts from T-ALL patients upon treatment [48].

## 3. Leukemic Niches in T-ALL

Many tissues and organs including the BM, thymus, spleen, lymph node (LN) and CNS are involved in the initiation and development of T-ALL. Thus, it is hard to define the “microenvironment” in terms of T-ALL [49,50]. Nonetheless, increasing evidence has revealed that the BM, thymic, splenic and CNS niches play key roles in the development of T-ALL [49,51,52,53,54,55]. Reciprocally, T-ALL cells highjack the healthy microenvironment by reshaping it into “pro-leukemic”, thereby promoting malignant progression and chemoresistance while disrupting normal functions [56,57,58,59,60,61].

### 3.1. Effects of Leukemic Niches on T-ALL Cells

#### 3.1.1. CXC Chemokine Ligand 12 (CXCL12)/CXC Chemokine Receptor 4 (CXCR4) Signaling

**Figure 2:**
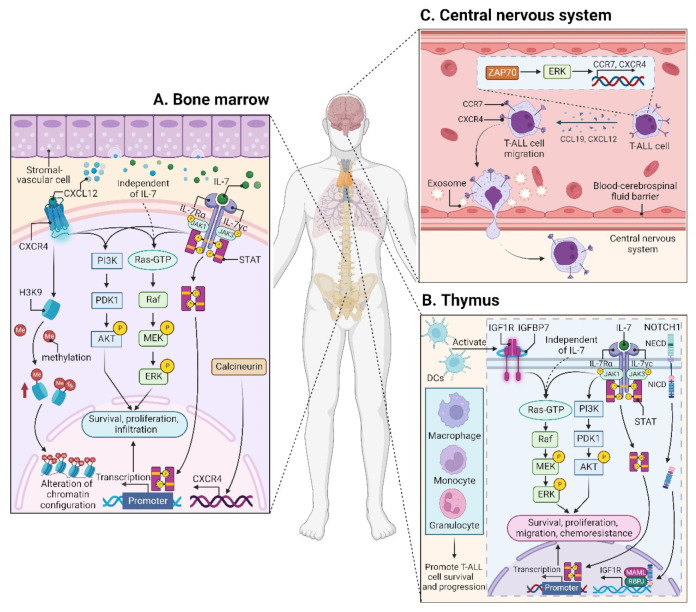
Microenvironments in T-ALL. (**A**) The interaction between CXCL12 and CXCR4 within the BM niche leads to activation of many signaling cascades, including PI3K-AKT and MEK-ERK pathways in T-ALL cells. CXCR12 also plays an epigenetic role by promoting H3K9 methylation in T-ALL cells. IL7 is produced by stromal cells in the BM (**A**) and thymus (**B**), facilitating the activation of JAK-STAT, PI3K-AKT, and MEK-ERK pathways in either an IL7-dependent or IL7-independent manner. (**B**) In the thymus, the high levels of IGF1R are supported by the activated NOTCH1 signaling. DCs support T-ALL growth via activating IGF1R and its downstream MAPK signaling; other myeloid cells including macrophages, monocytes, and granulocytes also support T-ALL survival and progression. In addition to IGF1R, IGFBP7, which binds to IGF1R, contributes to T-ALL by prolonging IGF1R activation. (**C**) The CCL19/CCR7 signaling is necessary and sufficient for CNS infiltration in T-ALL. Additionally, ZAP70-ERK-induced upregulation of CCR7 and CXCR4, as well as the exosomes isolated from T-ALL cells also confer an increased risk for CNS involvement. BM, bone marrow; CCL19, CC chemokine ligand 19; CCR7, CC chemokine receptor 7; CNS, central nervous system; CXCL12, CXC chemokine ligand 12; CXCR4, CXC chemokine receptor 4; DCs, dendritic cells; ERK, extracellular regulated protein kinase; H3K9, histone 3 lysine 9; IGF1R, insulin-like growth factor 1 receptor; IGFBP7, insulin-like growth factor binding protein 7; IL7, interleukin 7; JAK, Janus kinase; MAPK, mitogen-activated protein kinase; MEK, MAPK kinase; STAT, signal transducer and activator of transcription; ZAP70, zeta-chain-associated protein kinase 70.

CXCL12 (also termed stromal cell derived factor-1, SDF-1) is a chemotactic factor, and its receptor CXCR4 is a highly conserved G-protein-coupled seven transmembrane receptor. Since stromal-vascular BM niches secrete abundant CXCL12, whereas CXCR4 is primarily expressed on HSCs and malignant cells, the CXCL12/CXCR4 axis becomes a key pathway for both normal hematopoiesis and cancer cell homing to the BM [62,63,64,65]. Indeed, T-ALL cells express high surface levels of CXCR4 in a calcineurin-dependent manner, and CXCR4 silencing impairs migration and survival of leukemic cells, as well as T-LIC activity [66]. Likewise, CXCL12 deletion from vascular endothelial niches impedes T-ALL development [67]. Mechanistically, the interaction between CXCL12 and CXCR4 results in activation of many survival pathways including PI3K-AKT and mitogen-activated protein kinase (MAPK) signaling cascades in T-ALL cells [68] (Figure 2A). The CXCL12/CXCR4 signaling also mediates enhanced extramedullary infiltration and dissemination [69,70]. Therefore, the activation of CXCL12/CXCR4 signaling creates a sanctuary microenvironment for T-ALL cells and confers resistance to conventional therapies [68,71]. As such, inhibition of either the CXCL12/CXCR4 interaction or its downstream signaling is of therapeutic benefit in patients with chemoresistant T-ALL [72]. To date, CXCR4 antagonists such as AMD3465 or AMD3100 (plerixafor) were developed as a strategy to reduce leukemia burden and infiltration by disrupting the CXCL12/CXCR4 interaction [67,70]. Other novel CXCR4 inhibitors have also exhibited significant anti-leukemia activities in T-ALL [73,74,75]. For example, the CXCR4 antagonist BL-8040 is currently being tested in a Phase 2 clinical trial in combination with nelarabine for patients with relapsed or refractory (R/R) T-ALL (Table 1).

**Table 1:** Overview of agents evaluated in clinical trials including T-ALL.

Target	Treatment	Clinical Trials				Ref.
		Age	Phase	NCT No.	Status	
CXCR4	BL-8040 + nelarabine	≥18 y	2	NCT02763384	Recruiting	-
MEK	selumetinib + dexamethasone	Child, adult, older adult	1/2	NCT03705507	Recruiting	[76]
NOTCH1	MK0752	≥12 m	1	NCT00100152	Terminated	-
	PF-03084014	≥16 y	1	NCT00878189	Completed	[77,78]
	RO4929097 + dexamethasone	1–21 y	1	NCT01088763	Terminated	-
	BMS-906024 + dexamethasone	≥18 y	1	NCT01363817	Completed	-
	LY3039478 + dexamethasone	≥2 y	1/2	NCT02518113	Completed	[79]
BCL2	venetoclax + chemotherapy	Child, adult, older adult	2	NCT00501826	Recruiting	-
	venetoclax + navitoclax + chemotherapy	≥4 y	1	NCT03181126	Completed	[80]
	venetoclax + chemotherapy	≤25 y	1	NCT03236857	Recruiting	[81]
	venetoclax + vincristine liposomal	≥18 y	1/2	NCT03504644	Suspended	-
	venetoclax + low-intensity chemotherapy	≥18 y	1/2	NCT03808610	Recruiting	-
	venetoclax + navitoclax	≥18 y	1b/2	NCT05054465	Not yet recruiting	-
	venetoclax + azacitidine	15–65 y	2	NCT05149378	Recruiting	-
	venetoclax + navitoclax + chemotherapy	4–30 y	1/2	NCT05192889	Not yet recruiting	-
	venetoclax + ponatinib + mini-hyper CVD ^†^	≥18 y	2	NCT05268003	Recruiting	-
	venetoclax + azacitidine	≥15 y	2	NCT05376111	Recruiting	-
PI3K/mTOR	everolimus + hyper-CVAD ^††^	≥10 y	1/2	NCT00968253	Completed	-
	everolimus + reinduction chemotherapy	18 m–21 y	1	NCT01523977	Completed	-
	temsirolimus + chemotherapy	1–21 y	1	NCT01614197	Completed	-
	BEZ235	≥18 y	1	NCT01756118	Uk	[82]
	sapanisertib	≥18 y	2	NCT02484430	Active, not recruiting	-
	everolimus + chemotherapy	2–29 y	1	NCT03328104	Recruiting	-
CDK4/6	palbociclib + sorafenib, decitabine or dexamethasone	≥15 y	1	NCT03132454	Recruiting	-
	palbociclib + chemotherapy	≤21 y	1	NCT03515200	Terminated	-
	ribociclib + everolimus + dexamethasone	1–30 y	1	NCT03740334	Active, not recruiting	-
	palbociclib + chemotherapy	12 m–31 y	1	NCT03792256	Active, not recruiting	-
CD38	isatuximab + chemotherapy	≥16 y	2	NCT02999633	Terminated	[83]
	daratumumab + chemotherapy	1–30 y	2	NCT03384654	Active, not recruiting	-
	isatuximab + chemotherapy	28 d–17 y	2	NCT03860844	Recruiting	-
	daratumumab	≤39 y	2	NCT04972942	Not yet recruiting	-
	daratumumab + hyaluronidase	≥18 y	2	NCT05289687	Recruiting	-
CD38-CD3	XmAb18968 (bsAb)	≥18 y	1	NCT05038644	Recruiting	-
CD52	alemtuzumab + chemotherapy	≥15 y	1/2	NCT00061945	Completed	-
	alemtuzumab ± methotrexate, mercaptopurine	≤30 y	2	NCT00089349	Completed	[84]
	alemtuzumab ± cladribine	≥18 y	2	NCT00199030	Completed	-
	alemtuzumab + pentostatin	≥18 y	2	NCT00453193	Terminated	[85]

^†^ Mini-hyper CVD refers to low-intensity chemotherapy including cyclophosphamide, vincristine, and dexamethasone. ^††^ Hyper-CVAD refers to chemotherapy including cyclophosphamide, vincristine, adriamycin (doxorubicin), and dexamethasone. Note: BCL2, B-cell lymphoma 2; bsAb, bispecific antibody; CDK4/6, cyclin dependent kinase 4/6; CXCR4, CXC chemokine receptor 4; d, days; m, months; MEK, mitogen-activated protein kinase (MAPK) kinase; mTOR, mechanistic target of rapamycin kinase; ND, newly diagnosed; No., number; NOTCH1, notch receptor 1; PI3K, phosphatidylinositol 3-kinase; Ref, reference(s); T-ALL, T-cell acute lymphoblastic leukemia; Uk, unknown; y, years.

Very recently, CXCL12 was identified to promote histone 3 lysine 9 (H3K9) methylation in T-ALL cell lines and primary T-ALL cells rapidly, revealing for the first time a CXCL12-mediated epigenetic role in T-ALL cells [86] (Figure 2A). In fact, T-ALL was characterized by multiple epigenetic alterations, including DNA methyltransferases (DNMTs), enhancer of zeste homolog 2 (EZH2), histone deacetylases (HDACs), PHF6, etc., which were extensively reviewed [87,88,89,90]. Dysregulation of these modifiers not only reflects the complicated pathogenic mechanisms of T-ALL but also allows epigenetic treatment approaches, being of particular interest in T-ALL. The recent discoveries of euchromatic histone lysine methyltransferase 2 (EHMT2) and methyl-CpG binding domain protein 2 (MBD2) as novel epigenetic targets in T-ALL [91,92,93], together with CXCL12, will drive a new wave of epigenetic targeting molecules for further clinical investigation in T-ALL.

#### 3.1.2. Insulin-like Growth Factor 1 (IGF1)/IGF1 Receptor (IGF1R) Signaling

The leukemic niches are highly heterogeneous, being composed of different cell types, extracellular matrix, chemokines, and growth factors. Among them, dendritic cells (DCs) support T-ALL growth via activating IGF1R and its downstream MAPK signaling in the thymus; importantly, the IGF1R signaling is required for DC-mediated T-ALL survival in vitro [94]. Apart from DCs, myeloid cells, including macrophages, monocytes, and granulocytes, also support T-ALL survival and progression, both in vitro and in vivo; notably, some myeloid cells sensitize T-ALL cells to the IGF1R signaling, indicating that myeloid cells promote T-ALL progression at least in part by activating IGF1R signaling [95] (Figure 2B). Nonetheless, it remains unclear whether other signals are required to support T-ALL survival, since monocytes derived from peripheral blood mononuclear cells support T-ALL survival without activating IGF1R [95].

Of note, IGF1R is a NOTCH1 target, and the high levels of IGF1R in T-ALL cells are supported by the activated NOTCH1 signaling [96,97]. Thus, the inhibition of IGF1R signaling, either by genetic or pharmacologic means, compromises T-LIC activity, suggesting that the effects of NOTCH1 on the T-LIC activity may be mediated in part by IGF1R signaling [96]. Later on, the same group revealed that T-ALL generated from fetal liver (FL) and adult BM have a dramatic difference in their T-LIC activity, i.e., that FL-derived leukemia with robust NOTCH1-driven autocrine IGF1 signaling exhibits 2-log lower T-LIC activity compared to BM-derived leukemia [98]. This observation led the authors to evoke fetal-like programs in BM-derived T-ALL. Of particular interest, re-engagement of IGF1 signaling in mouse T-ALL and patient-derived tumor xenograft (PDX) models effectively limits T-LIC activity, presumably by drawing quiescent T-LICs into cycle, thereby leading to T-LIC depletion [98]. Notably, the FL- and BM-derived leukemias might closely resemble pediatric and adult leukemias, respectively. With this in mind, this research, together with the previous one [96], highlights the distinct roles of IGF1 signaling in different age-related programs.

In addition to the IGF1/IGF1R axis, insulin-like growth factor binding protein 7 (IGFBP7), which binds to IGF1R, plays an oncogenic role in T-ALL by promoting the perdurance of IGF1R and prolonging IGF1R activation [99] (Figure 2B). Therefore, the inhibition of IGF1R could overcome IGFBP7-induced resistance to vincristine in T-ALL [100].

#### 3.1.3. Interleukin (IL7)/IL7 Receptor (IL7R) Signaling

IL7 is produced by stromal cells in the BM and thymus. It binds to IL7R, a heterodimer composed of an IL7Rα chain and a γ_c_ chain [101]. The IL7/IL7R signaling is critical to the survival and proliferation of thymocytes, and the tight regulation of IL7 and IL7Rα is essential for T-cell development. Hence, it is not surprising that aberrant expression or dysfunction of the IL7/IL7R axis contributes to the pathogenesis of T-ALL [102,103]. Several lines of evidence have demonstrated that the somatic gain-of-function mutation in *IL7Ra* exon 6 is a known driver of T-ALL, occurring in roughly 9% of pediatric and 12% of adult T-ALL cases [104,105,106,107,108,109]. Mechanistically, *IL7Ra* mutations induce activation of Janus kinase (JAK)-signal transducer and activator of transcription (STAT), PI3K-AKT, and MAPK kinase (MEK)-extracellular regulated protein kinase (ERK) pathways [76,104,110]. Overexpression of wild-type IL7Rα also promotes T-cell tumorigenesis via JAK-STAT, PI3K-AKT, and cell-cycle-related signaling, even in the absence of *IL7Rα* mutational activation [111]. Moreover, high levels of IL7Rα expression lead to increased T-LIC activity mediated by the activating mutations in *NOTCH1* [112] or the loss-of-function mutations in *DYNAMIN2* [113]. These findings pinpoint that not only mutational activation of *IL7Rα* but also high IL7Rα levels are oncogenic in T-ALL (Figure 2B). In addition to IL7Rα, mutations in other components of the IL7R-mediated signaling cascade (e.g., JAK1, JAK3, STAT5) were also identified as critical drivers for T-ALL [106,108,114,115,116,117]. These mutations and the resulting aberrant signaling provide a therapeutic window of opportunity [108]. Indeed, combined therapy with MEK and PI3K-AKT inhibitors has synergistic cytotoxic effects in leukemic cells from pediatric T-ALL patients carrying IL7R signaling mutations [108]. The JAK1/2 inhibitor ruxolitinib is also promising to restore steroid sensitivity in pediatric T-ALL cases [110]. However, none of the pediatric T-ALL PDX samples respond to single-agent ruxolitinib in the absence of IL7, whereas MEK inhibitors enhance steroid responsiveness in both IL7-dependent and IL7-independent steroid-resistant pediatric T-ALL samples, highlighting the central role for MAPK-ERK signaling in steroid resistance [76]. In contrast to pediatric T-ALL, data are scarce in adult T-ALL. In a large cohort of adult T-ALL cases, patients with IL7R signaling mutations are slow-responders to chemotherapy and do not benefit from allogeneic hematopoietic stem-cell transplantation (HSCT) [109]. This finding suggests that the mutational status of the IL7R pathway could influence the use of HSCT, requiring optimization of therapeutic strategies to improve clinical outcome in adult T-ALL.

#### 3.1.4. CC Chemokine Ligand 19 (CCL19)/CC Chemokine Receptor 7 (CCR7) Signaling

Patients with T-ALL are at a high risk of CNS relapse [21], which requires intensified intrathecal chemotherapy and systemic administration of CNS-penetrating therapeutics. Unfortunately, the mechanisms accounting for the infiltration of T-ALL cells into CNS remain incompletely understood, and little is known about their crosstalk.

The CCL19/CCR7 signaling was identified as a key CNS entry signal, which is both necessary and sufficient for T-ALL cells targeting the CNS. Silencing either CCR7 or CCL19 specifically inhibits CNS infiltration [118]. Furthermore, the BM infiltration constitutes a prerequisite for CNS pathology in T-ALL via the CXCR4-mediated signaling [70]. In this sense, CNS invasion by T-ALL cells is very likely attributed to the BM microenvironmental alterations. Interestingly, both CCR7 and CXCR4 are upregulated by zeta-chain-associated protein kinase 70 (ZAP70) via the activation of ERK signaling, and high expression of ZAP70-CCR7 confers an increased risk for CNS involvement in T-ALL patients [119] (Figure 2C). Therefore, therapeutic monoclonal antibodies (mAbs) targeting CCR7 not only display a strong in vitro complement-dependent cytotoxicity (CDC) and an in vivo anti-tumor activity, but also show efficacy in eradicating leukemic cells from LN and CNS [120]. In addition to the CCR7-mediated CNS infiltration, the exosomes isolated from a T-ALL cell line P12 but not the B-cell acute lymphoblastic leukemia (B-ALL) cell lines facilitate CNS invasion across the blood–cerebrospinal fluid (CSF) barrier without disrupting the barrier integrity [121] (Figure 2C), highlighting the contribution of T-ALL-derived exosomes to CNS infiltration, although further studies are needed to clarify the molecular mechanisms.

It is worth noting that the CNS-resident leukemic cells are bathed in CSF, a harsher microenvironment with lower oxygen and nutrients than the BM [122]. In this circumstance, leukemic cells need to adapt their metabolism to survive and proliferate. Indeed, the B-ALL cells that infiltrate the CNS enhance hypoxic adaptation by upregulating vascular endothelial growth factor A [123] or increase metabolic adaptation by altering metabolic profiling and pathways [124,125]. Intriguingly, B-ALL cells residing in the CNS and BM have distinct metabolic signatures [124] and mRNA translation [125], highlighting the metabolic plasticity of leukemic cells in different microenvironments, and provide novel therapeutic strategies to target metabolism in the setting of disseminated hematological malignancies. Nevertheless, little is known about how T-ALL cells metabolically adapt to the CNS microenvironment, which warrants further study.

### 3.2. Microenvironmental Alterations

#### 3.2.1. BM Microenvironment

**Figure 3:**
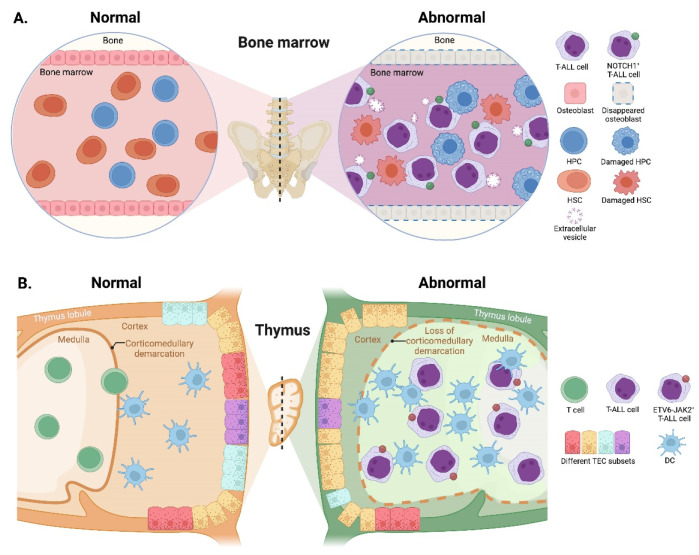
Reciprocal effects of T-ALL cells on microenvironments. (**A**) In the BM, accumulated T-ALL burden leads to a complete loss of osteoblasts and induces impairment of HSCs and HPCs. T-ALL-produced extracellular vesicles also disturb the quiescence and maintenance of HSCs and HPCs, thus, inducing exhaustion of the normal HSCs and HPCs. (**B**) In the thymus, the invasion of T-ALL cells impairs the normal corticomedullary demarcation accompanied by strong cortical reduction and medullary expansion. The proportions of TEC subsets and the number of DCs are also remarkably altered. HPCs, hematopoietic progenitor cells; HSCs, hematopoietic stem cells; TEC, thymic epithelial cell.

Modification of the BM microenvironment by leukemia cells was well-characterized in B-ALL and AML [126,127,128], whereas the research on the alterations of the BM microenvironment by T-ALL cells is very limited. In 2016, an elegant work reported that an accumulated T-ALL burden within the BM leads to rapid and selective remodeling of the endosteal space, resulting in a complete loss of mature osteoblasts and impairment of normal HSCs [56]. The same conclusion was reached by another group, who additionally found that T-ALL cells suppress osteoblasts and hematopoiesis via activation of the NOTCH1 signaling, since the NOTCH1 blockade attenuates suppression of osteoblasts and HSCs [57]. Very recently, scientists discovered that T-ALL-produced extracellular vesicles can also disturb the quiescence and maintenance of HSCs and HPCs in the murine BM environment, thereby inducing exhaustion of the healthy HSCs and HPCs to compromise the hematopoietic system balance [58] (Figure 3A). Although these works have revealed certain effects of T-ALL cells on BM remodeling and dysfunction, it remains unclear whether additional mechanisms contribute to T-ALL-induced BM environmental alterations, a critical topic requiring further investigation.

#### 3.2.2. Thymic Microenvironment

The thymus is a conserved primary lymphoid organ where progenitors from the BM commit to T-cell lineage development. Thus, thymocytes were long thought to be short-lived cells with no self-renewal capacity. However, two independent groups have updated the notion with the fact that the thymus is capable of sustaining T-cell development and export independently from BM contribution, a state termed as thymus autonomy [129,130]. However, thymus autonomy must be tightly regulated, as prolonged autonomy allows profound alterations in the thymic microenvironment, contributing to the initiation and propagation of T-ALL [131]. In this regard, T-ALL is proposed as a consequence of thymus autonomy [132].

Reciprocally, upon the emergence of T-ALL cells in the thymus, their invasion exerts an impact on the differentiation and composition of thymic epithelial cells (TECs) before expanding. The normal corticomedullary demarcation is lost accompanied by strong cortical reduction and medullary expansion during the onset of ETS variant transcription factor 6 (ETV6)-JAK2-induced T-ALL in mice; further gene expression analysis reveals remarkable alterations in TEC subset proportions and an increase in DCs [59] (Figure 3B). These microenvironmental alterations further lead to substantial changes in the proteins expressed in TECs, such as forkhead box N1 and lymphotoxin-β receptor [59,60], which in turn, affect the leukemogenesis of T-ALL.

#### 3.2.3. Splenic Microenvironment

The spleen is a common extramedullary site of leukemia, and splenomegaly is associated with poor clinical outcome in many subtypes of leukemias including T-ALL [133,134,135]. However, the spleen was largely ignored as a tumor microenvironment site due to the difficulties in obtaining biopsy samples from patients. In a previous work, the leukemia-associated macrophages within the spleen were revealed to recruit T-ALL cells potently and stimulate their proliferation [53]. Further investigation demonstrated that the mice transplanted with the spleen-resident T-ALL cells exhibit a short life span compared to those transplanted with T-ALL cells from the BM, suggesting an increased potency in T-ALL cells induced by the splenic microenvironment [54]. There is even evidence that splenectomies either before or after the injection of T-ALL cells prolong the survival of mice but do not inhibit the development of T-ALL [54]. In contrast, removal of the spleen in a genetic mouse model of delta-like canonical Notch ligand 4 (DLL4)-driven T-ALL fully protects against leukemia development [55], indicating a crucial role of the spleen in DLL4-driven T-ALL.

Recently, one study has revealed that the splenic microenvironment reduces apoptotic sensitivity of T-ALL cells to the inhibitors of B-cell lymphoma 2 (BCL2); interestingly, a small population of CD34^+^ cells are enriched in the splenic niche following the treatment of BCL2 inhibitors [61], suggesting that the spleen provides a sanctuary microenvironment for the immature T-ALL subset. More strikingly, the therapeutic pressure of BCL2 inhibitors induces transcriptional remodeling of the residual T-ALL cells within the splenic niche, revealing the spleen as a potential site of chemoresistance and disease relapse [61]. However, whether the T-ALL remodeling is driven by the splenic niche, or how the engraftment of T-ALL affects the splenic microenvironment has yet to be addressed.

## 4. Preclinically- and Clinically-Evaluated Precision Medicine for T-ALL

The high-dose multiagent chemotherapies have so far remained the frontline treatment for patients with T-ALL [136]. Over 80% of children have benefited from the contemporary regimens, but they always suffer from short- and long-term toxicities. What is worse, nearly half of adult patients with T-ALL are insensitive to chemotherapies, resulting in R/R cases. Therefore, a huge effort should be made to optimize stratification and therapy at the initial diagnosis, the best time for therapeutic intervention. Due to advances in understanding the molecular pathogenesis of T-ALL, many novel stratified markers and targeted medicines have emerged over the past decades. The most promising therapeutics are immunotherapies, which are increasingly being incorporated into clinical trials and daily clinical practice. Since the current frontline treatment and chimeric antigen receptor (CAR)-based immunotherapy for T-ALL were recently reviewed [1,137], we, here, exclusively discussed the small-molecule inhibitors and mAbs targeting aberrant pathways or themselves that are associated with T-LICs and leukemic niches, with the other agents omitted in this review unless specified otherwise.

### 4.1. Agents Targeting Aberrant Pathways

#### 4.1.1. NOTCH1 Signaling

NOTCH1 signaling is an attractive therapeutic target for T-ALL due to its essential roles in T-ALL initiation and progression. Therefore, its inhibition via GSIs was investigated as a potential targeted therapeutic in preclinical studies [138], followed by a series of clinical trials (Table 1) [78,79]. Despite a hint of clinical efficacy, GSIs were not applied to clinical practice. One major reason is that GSIs have activity against T-ALL with *NOTCH1* mutations but not those with *PTEN* deficiency and activation of PI3K-AKT signaling [30], as well as constitutive MYC expression [139]. Another key problem is the occurrence of gastrointestinal toxicity and unsatisfactory clinical trials caused by the use of broad-spectrum GSIs [79,140,141]. In this context, selective targeting of the certain components of γ-secretase complexes [142] or putative responders [139] may improve antileukemic efficacy while sparing patients from excessive toxicities.

In addition to GSIs, anti-NOTCH1 mAbs against the negative regulatory region (NRR) act as potent inhibitors of the NOTCH1 signaling in T-ALL cells [143,144]. OMP-52M51, a novel mAb targeting NRR, not only delays engraftment of T-ALL cells in xenografts derived from poor responders or relapsed patients but also impairs the functional activity of T-LICs; more importantly, OMP-52M51 enhances the in vivo effects of dexamethasone, highlighting the therapeutic potential of anti-NOTCH1 mAbs in combination with steroids [145].

#### 4.1.2. BCL2 Signaling

BCL2, a key regulator of the apoptotic pathway, has emerged as another attractive molecular target in T-ALL due to its high expression in T-ALL cells [146]. Navitoclax (ABT-263) is the first generation of the BCL2 inhibitor, whereas venetoclax (ABT-199) is a selective BCL2 inhibitor [147]. The antileukemic effects of venetoclax in T-ALL, either as a monotherapy or in combination with chemotherapy, were evaluated experimentally [146] and clinically (Table 1). Two recent case reports demonstrated complete remissions (CR) in patients following the treatment with venetoclax in combination with nelarabine or decitabine [148,149]. Nonetheless, the results from a Phase 1 trial in R/R ALL (NCT03181126) showed that one T-ALL patient achieved CR following the chemotherapy in combination with navitoclax and venetoclax but eventually relapsed [150]. One possible mechanism might be the adaptative chemoresistance caused by other activated pro-survival factors. Indeed, combination therapies targeting different BCL2-family members, including BCL2, MCL1 apoptosis regulator, and/or BCL2-like 1, have shown synergistic antileukemic effects in T-ALL cells, both in vitro and in vivo [151,152]. These findings provide a novel strategy to improve the long-term therapeutic efficacy of BCL2 inhibition in T-ALL.

#### 4.1.3. JAK-STAT Signaling

Active signaling via JAK1/2 is widely reported in T-ALL cells and leukemic niches, making JAK1/2 inhibitor ruxolitinib a promising agent. However, in preclinical PDX models of T-ALL, treatment with ruxolitinib as a single agent exhibits dramatic efficacy but fails to achieve CR [153]. Further results from a Phase 1/2 open-label trial in adult T-cell leukemia (ATL) (NCT01712659) showed that monotherapy with ruxolitinib is safe but insufficient to produce clinical benefit in patients with indolent ATL [154]. These results call for the need for multiagent combination. Intriguingly, the combination of ruxolitinib with doxorubicin or vincristine has no synergistic but rather an antagonistic effect on T-ALL cells, whereas combining ruxolitinib with dexamethasone significantly increases apoptosis of T-ALL cells and reduces the leukemic burden in T-ALL PDX models [155], in line with previous studies demonstrating increased steroid sensitivity when combined with ruxolitinib [110,156]. The combination of ruxolitinib and venetoclax is also highly active preclinically and has promising clinical effects in two patients with refractory T-cell prolymphocytic leukemia (T-PLL) [157]. Although ruxolitinib was not clinically evaluated in a T-ALL setting, we could at least obtain some clues from the clinical trials for other T-cell malignancies.

#### 4.1.4. PI3K-AKT-mTOR Signaling

Inhibitors that target PI3K-AKT-mTOR pathway were largely evaluated under clinical trials for T-ALL (Table 1). However, the complex interplay between NOTCH1-PI3K-AKT and PTEN-PI3K-AKT signaling makes the targeted therapy much trickier. Treatment with GSIs in PTEN-deficient cells results in hyperactivation of AKT [158]. Dual inhibition of PI3K and mTOR pathways triggers NOTCH1-MYC activity [159]. These discoveries highlight the necessity of combination therapies by concurrently blocking different pathways or a common downstream effector in T-ALL. Very recently, one group determined the heterogeneity and cellular plasticity of R/R T-ALL cells carrying activating *NOTCH1* mutations at a single-cell resolution [160]. They revealed two highly distinct stem-like populations: fast-cycling cells with NOTCH1 activation and slow-cycling cells with PI3K activation independent of NOTCH1. This study defines a new cellular state that characterizes treatment failure in *NOTCH1*-mutated R/R T-ALL with GSIs, shedding light on developing more effective treatments for R/R T-ALL by targeting a specific subset.

#### 4.1.5. CDK4/6-Mediated Signaling

Both CDK4 and CDK6 are the targets of NOTCH1 signaling and contribute to the deregulated cell-cycle progression in T-ALL cells [161]. Thus, inhibition of CDK4/6 activity efficiently suppresses T-ALL progression in vivo but most likely not target T-LICs, because interruption of drug administration leads to disease relapse [162]. Another group further discovered that CDK6-mediated suppression of CD25 is required for initiation of *Notch1*-induced T-ALL; notably, CD25^+^ T-ALL cells are sensitive to CDK6 inhibition in vivo, whereas CD25^−^ T-ALL cells are insensitive even though CDK6 is expressed [17]. This finding implies the selective activity of CDK4/6 inhibitors (CDKis) in a certain T-ALL subset. In addition, prolonged monotherapy of CDKis induces resistance in T-ALL [163], emphasizing the importance of combining CDKis with conventional chemotherapy. To this end, a number of clinical trials are currently underway to test the efficacy of combining CDKis with different agents in patients with T-ALL (Table 1).

#### 4.1.6. Other Signaling Pathways

One recent study has identified a novel small-molecule inhibitor Dynole 34-2, which is a specific and potent inhibitor of Dynamin. Dynole 34-2 not only impairs T-LIC activity but also sensitizes them to chemotherapy. More essentially, Dynole 34-2 exhibits efficacy against multiple niche signals in T-LICs including IL-7, NOTCH1, etc. [35]. This discovery provides a significant advance in developing therapeutic strategies by targeting T-LICs concurrently with multiple microenvironmental signals.

Other pathways required for T-ALL also include the overactive kinase signals, such as tyrosine kinase signaling. Dasatinib is a potent tyrosine kinase inhibitor (TKI) that has shown satisfactory efficacy and acceptable safety in T-ALL, alone or in combination with chemotherapy [164,165,166,167,168]. However, TKIs are not very potent in eradicating the LSCs in chronic myeloid leukemia [169,170]. In this case, it would be interesting to evaluate the effect of Dasatinib on T-LICs.

### 4.2. Antibody-Based Therapy

#### 4.2.1. CD38 mAbs

T cells can be activated via T cell receptor or by triggering multiple cell surface molecules including CD38. Although it is unknown whether T-LICs are positive or negative for CD38, blasts from patients with T-ALL have robust surface expression of CD38 at the time of diagnosis, 1 month post induction, and relapse, making it an ideal target for T-ALL patients who relapse or do not respond to conventional chemotherapies [171,172]. Daratumumab, a fully human mAb against CD38, was identified to be highly effective in T-ALL PDX models (14 out of 15), and the only PDX model that failed to respond to daratumumab showed low expression of CD38 [172]. More essentially, daratumumab can effectively eradicate minimal residual disease in preclinical models of pediatric T-ALL and high-risk advanced relapse T-ALL [173,174], providing compelling evidence for the potential clinical efficacy of daratumumab in T-ALL. As such, clinical trials testing the efficacy of daratumumab in T-ALL are currently being evaluated, and the same is true for another anti-CD38 mAb, isatuximab [83] (Table 1). Very recently, the preliminary results released from a phase 2 trial of daratumumab in combination with chemotherapy (NCT03384654) are encouraging, with an overall response rate of 83.3% in children and 60% in young adults with R/R T-ALL [175]. In spite of this, a recent clinical report indicated that not all patients with R/R T-ALL responded to daratumumab administration [176]. One major reason is the loss of CD38, which could be overcome by monitoring CD38 during treatment or using other antibodies by targeting different epitopes or molecules.

Indeed, combining daratumumab with anti-CD47 antibodies significantly enhances antibody-dependent cellular phagocytosis in both in vitro and in vivo settings [177]. Of note, CD47 blockade alone is sufficient to prolong survival of random de novo T-ALL PDX models, since all mice treated with anti-CD47 antibodies showed long-term survival with none of the mice developing leukemia, whereas monotherapy with daratumumab only displayed prolonged survival in 67% of the cases and the combined treatment had no further survival benefit. This finding indicates that targeting CD47 alone is highly efficient, no matter with or without the addition of daratumumab in this setting [177]. Nevertheless, a combined therapy with daratumumab and anti-CD47 antibodies significantly prolongs survival in R/R T-ALL PDX models [177]. Apart from the combination of mAbs, XmAb18968, a bispecific antibody targeting both CD3 and CD38, is also under evaluation in a Phase 1 trial for R/R T-ALL (Table 1).

#### 4.2.2. CD52 mAbs

CD52 is widely expressed on normal and malignant B and T cells, and its function remains largely unknown. Alemtuzumab is a humanized anti-CD52 mAb that causes cell death by antibody-dependent cell mediated cytotoxicity (ADCC), CDC, and apoptosis upon binding to CD52 [178]. Although different clinical trials have tested the efficacy of alemtuzumab for T-cell malignancies including T-ALL [84,85,179,180] (Table 1), the activity of single-agent alemtuzumab is limited in children with R/R T-ALL based on a Phase 2 study (NCT00089349) [84]. Additionally, no response was observed in adults with relapsed T-ALL when combining alemtuzumab with pentostatin (NCT00453193) [85]. As a consequence, no new trials targeting CD52 have been initiated for T-ALL.

#### 4.2.3. IL7Rα mAbs

Apart from the inhibitors of IL7R-mediated signaling as aforementioned, a more direct strategy is to explore antibody-based treatment by targeting IL7Rα itself. Notably, a fully human anti-IL7Rα antibody (B12) that recognizes both the wild-type form and different gain-of-function mutated variants was generated using combinatorial phage-display libraries and antibody reformatting [181]. B12 not only promotes T-ALL cell death in vitro and delays T-ALL development in vivo, but it also sensitizes T-ALL cells to dexamethasone. More importantly, B12 exhibits a remarkably fast internalization with substantial trafficking into lysosomes, making it an ideal deliverer for toxin conjugates. Recently, another two new chimeric mAbs against human IL7Rα (4A10 and 2B8) that target non-overlapping IL7Rα epitopes were reported [182]. Both 4A10 and 2B8 mediate increased ADCC against patient-derived T-ALL cells and lead to effective anti-leukemia responses in vivo. Unlike B12, 4A10 and 2B8 cannot induce rapid internalization, but this feature would be desirable for promoting ADCC. Although anti-IL7Rα mAbs were only investigated in preclinical models, future clinical evaluation in T-ALL is eagerly anticipated.

## 5. Challenges and Future Perspectives

Over the past few decades, numerous attempts were made to dissect the initiating steps of T-ALL in xenografts; however, many critical challenges remain. On one hand, enforced expression or silence of a certain gene cannot fully cover all the involved signals during leukemogenesis. On the other hand, along with disease progression, leukemic cells remain in contact with multiple microenvironments (e.g., the thymus, BM, spleen, and CNS), which exert profound impacts on their intrinsic behaviors and responses to chemotherapeutics simultaneously. In this context, single-cell sequencing might be an ideal approach to delineate the emergence and formation of T-LICs. Indeed, two groups have recently elucidated the heterogeneity of T-ALL at a single-cell level, providing an exciting strategy to identify molecular targets for individualized therapy [183,184]. In addition, establishment of models with biologically-defined subtypes such as pediatric and adult T-ALL is also required to decipher the genetic/epigenetic code of T-LICs and leukemic niches in different age groups.

Another major challenge is that most patients with T-ALL receive similar therapies regardless of the underlying pathogenesis. Therefore, future treatment should rely on the well-tailored therapy specific to the individual characteristics of each patient by targeting T-LICs or leukemic niches. Thanks to the next-generation sequencing technology and the development of immunotherapy, we are now entering an exciting era of personalized therapy. However, unlike other leukemic contexts where chemo-free regimens have emerged, it is hard to develop chemo-free precision medicine for T-ALL due to its high heterogeneity and complex pathogenesis. For example, GSIs, ruxolitinib and alemtuzumab as monotherapy have shown very limited responses in T-ALL. On the other hand, despite the advances in understanding the genetic landscape of T-ALL, the translation of novel targeted therapies into clinical practice has remained in its infancy. Therefore, the future direction for treatment should focus on the incorporation of novel targeted agents into conventional regimens, allowing synergistical activities on leukemia cells by disrupting multiple pathogenic pathways at the initial stage.

Finally, many key factors and pathways for T-ALL are required for normal hematopoiesis. The best way to reduce excessive toxicity is to develop more selective inhibitors. One representative example is the GSIs, which are not specific to NOTCH1, as they also affect cleavage of other proteins [185]. In this regard, identification of all the components of γ-secretase complexes and their respective substrates are of particular importance for targeted therapeutic development. In addition, CAR-based therapy provides an alternative strategy to target leukemia cells, but there are many obstacles to their use including cytokine release syndrome, fratricide killing, and early exhaustion [137]. In contrast, developing mAbs specifically against T-LICs or leukemic niches (e.g., anti-NOTCH1 and anti-IL7Rα), along with chemotherapy, will open a brand-new era for the treatment of T-ALL in the very near future.

## 6. Conclusions

During the initiation and progression of T-ALL, the contribution of T-LICs and leukemic niches are not fragmented from each other. T-LICs are formed and maintained within the leukemic niches, which in turn, affect lineage plasticity and stemness. Their behaviors and interactions further evolve into a more complicated state under stressful conditions such as chemotherapy. Thus, many questions remain to be further addressed. For example, how do T-LICs and leukemic niches alter upon treatment? Why do leukemic cells quit the thymus to spread throughout the body? What events determine the BM as a warm nest for T-ALL cells? What are the long-term effects of chemotherapy on thymic, splenic, and BM niches after the elimination of leukemic cells? These answers will lead us to a comprehensive understanding of the entire landscape of the initiation of T-LICs, as well as the reciprocal relations between T-ALL cells and leukemic niches, thus, providing evidence-based guidelines for targeted therapy.

## Abbreviations

ADCC	antibody-dependent cell mediated cytotoxicity
AKT	AKT serine/threonine kinase
AML	acute myelocytic leukemia
ATL	adult T-cell leukemia
B-ALL	B-cell acute lymphoblastic leukemia
BCL2	B-cell lymphoma 2
BM	bone marrow
CAR	chimeric antigen receptor
CCL19	CC chemokine ligand 19
CCR7	CC chemokine receptor7
CDC	complement-dependent cytotoxicity
CDK4/6	cyclin dependent kinase 4/6
CDKis	CDK4/6 inhibitors
CNS	central nervous system
CR	complete remissions
CSF	cerebrospinal fluid
CXCL12	CXC chemokine ligand 12
CXCR4	CXC chemokine receptor 4
DCs	dendritic cells
DLL4	delta-like canonical Notch ligand 4
DN	CD4^−^CD8^−^ double-negative
DNMTs	DNA methyltransferases
DP	CD4^+^CD8^+^ double-positive
EHMT2	euchromatic histone lysine methyltransferase 2
ERK	extracellular regulated protein kinase
ETV6	ETS variant transcription factor 6
EZH2	enhancer of zeste homolog 2
FL	fetal liver
GSIs	γ-secretase inhibitors
H3K9	histone 3 lysine 9
HDACs	histone deacetylases
HIF1α	hypoxia inducible factor 1α
HPCs	hematopoietic progenitor cells
HSCs	hematopoietic stem cells
HSCT	hematopoietic stem-cell transplantation
IGF1(R)	insulin-like growth factor 1 (receptor)
IGFBP7	insulin-like growth factor binding protein 7
IL7(R)	interleukin 7 (receptor)
JAK	Janus kinase
KARS (Kras)	KRAS proto-oncogene
KLF4	Krüppel-like factor 4
LIC(s)	leukemia-initiating cell(s)
LMO1/2 (Lmo1/2)	LIM domain only 1/2
LN	lymph node
LSCs	leukemia stem cells
mAbs	monoclonal antibodies
MAPK	mitogen-activated protein kinase
MBD2	methyl-CpG binding domain protein 2
MEK	MAPK kinase
mTOR(C1)	mechanistic target of rapamycin kinase (complex 1)
MYC (Myc)	MYC proto-oncogene
NOTCH1	notch receptor 1
NRR	negative regulatory region
PDX	patient-derived tumor xenograft
PHF6	plant homeodomain factor 6
PI3K	phosphatidylinositol 3-kinase
PKCθ	protein kinase C theta
PTEN (Pten)	phosphatase and tensin homolog
R/R	relapsed/refractory
ROS	reactive oxygen species
RUNX	runt-related transcription factor
SDF-1	stromal cell derived factor-1
STAT	signal transducer and activator of transcription
TAL1 (Tal1)	T-cell acute lymphocytic 1 (TAL) basic helix-loop-helix (bHLH) transcription factor 1
T-ALL	T-cell acute lymphoblastic leukemia
TECs	thymic epithelial cells
TKI(s)	tyrosine kinase inhibitor(s)
T-LICs	T-ALL LICs
T-PLL	T-cell prolymphocytic leukemia
ZAP70	zeta-chain-associated protein kinase 70
